# Combining micro-RNA and protein sequencing to detect robust biomarkers for Graves’ disease and orbitopathy

**DOI:** 10.1038/s41598-018-26700-1

**Published:** 2018-05-30

**Authors:** Lei Zhang, Giulia Masetti, Giuseppe Colucci, Mario Salvi, Danila Covelli, Anja Eckstein, Ulrike Kaiser, Mohd Shazli Draman, Ilaria Muller, Marian Ludgate, Luigi Lucini, Filippo Biscarini

**Affiliations:** 1 Cardiff University, School of Medicine, Division of Infection & Immunity, Cardiff, UK; 2 Parco Tecnologico Padano, Bioinformatics Unit, Lodi, Italy; 3 Università degli Studi di Milano, Fondazione Ca’ Granda IRCCS, Department of Clinical Sciences and Community Health, Milan, Italy; 4 University Hospital Essen/University of Duisburg-Essen, Department of Ophthalmology, Essen, Germany; 5 Università Cattolica del Sacro Cuore, Department for Sustainable food process, Piacenza, Italy; 6 CNR (National Council for Research), Institute of Biology and Biotechnology in Agriculture (IBBA), Milan, Italy

## Abstract

Graves’ Disease (GD) is an autoimmune condition in which thyroid-stimulating antibodies (TRAB) mimic thyroid-stimulating hormone function causing hyperthyroidism. 5% of GD patients develop inflammatory Graves’ orbitopathy (GO) characterized by proptosis and attendant sight problems. A major challenge is to identify which GD patients are most likely to develop GO and has relied on TRAB measurement. We screened sera/plasma from 14 GD, 19 GO and 13 healthy controls using high-throughput proteomics and miRNA sequencing (Illumina’s HiSeq2000 and Agilent-6550 Funnel quadrupole-time-of-flight mass spectrometry) to identify potential biomarkers for diagnosis or prognosis evaluation. Euclidean distances and differential expression (DE) based on miRNA and protein quantification were analysed by multidimensional scaling (MDS) and multinomial regression respectively. We detected 3025 miRNAs and 1886 proteins and MDS revealed good separation of the 3 groups. Biomarkers were identified by combined DE and Lasso-penalized predictive models; accuracy of predictions was 0.86 (±0:18), and 5 miRNA and 20 proteins were found including Zonulin, Alpha-2 macroglobulin, Beta-2 glycoprotein 1 and Fibronectin. Functional analysis identified relevant metabolic pathways, including hippo signaling, bacterial invasion of epithelial cells and mRNA surveillance. Proteomic and miRNA analyses, combined with robust bioinformatics, identified circulating biomarkers applicable to diagnose GD, predict GO disease status and optimize patient management.

## Introduction

Prevention is better than cure, a maxim which applies to all diseases and has led to efforts to identify biomarkers defined by the National Institutes of Health as “a characteristic that is objectively measured and evaluated as an indicator of normal biologic processes, pathogenic processes, or pharmacologic responses to a therapeutic intervention”^1^. The discovery of biomarkers is one example of the translation of fundamental research into clinical applications. On one hand it helps elucidate disease aetiology and mechanisms, on the other hand biomarkers can be useful clinical tools (diagnosis, prognosis, therapy indications etc)^2,3^. Initial attempts to discover biomarkers were based on immunoassays (e.g. ELISA) or hybridization microarrays^4^. ‘Omics data from high-throughput next-generation sequencing (NGS) technology are now increasingly used for biomarker screening^5,6^. Biomarkers can be detected from several tissues and fluids, such as urine^7^, saliva^8^, swabs^9^, and blood^10,11^. Blood biomarkers were originally screened in relation to blood and cardiometabolic diseases, but also found applications in systemic conditions like cancer, infections, degenerative diseases and autoimmune conditions^12–15^.

Graves’ orbitopathy (GO) is an eye disease occurring mainly in patients with Graves’ disease (GD), in which autoantibodies (TRAB) to the thyrotropin receptor (TSHR) cause hyperthyroidism. GD is associated with considerable genetic susceptibility^16^, including polymorphisms in the TSHR and CD40 genes, with the latter leading to cytokine imbalance in the thyroid^17^. In GO, inflammation with subsequent remodelling and expansion of orbital tissues leads to proptosis accompanied by corneal exposure, diplopia etc., and may even result in blindness. Moderate GO occurs in 30–50% of GD patients, but the most severe eye condition is found in 5% of patients^18,19^. One of the challenges has been to identify which GD patients are most likely to develop GO: elevated serum TRAB have been correlated to the severity of GO^20^, and high TRAB combined with low/absent autoantibodies to thyroid peroxidase potentially identify such patients^21^. Blood biomarkers for GD and GO may facilitate the diagnosis and follow-up (i.e. monitoring progression) of patients, identifying individuals most likely to develop the eye condition. Currently, the diagnosis of GD relies on examination of the thyroid, measurement of serum thyroid hormones concentrations and TRAB assay, while predicting the progression from GD to GO is imperfect and relies mostly on clinical signs. Biomarkers could complement diagnosis and monitoring of this disease by: (i) increasing accuracy, (ii) reducing costs, (iii) speeding up time to diagnosis. Additionally, the loss of immune-tolerance mechanisms in GD/GO is poorly understood.

Circulating proteins and microRNA (miRNA) are the two most widely used classes of blood biomarkers for clinical research; for miRNA, this is linked to their emerging mechanistic role in specific disorders, e.g. metabolic and cardiovascular diseases^22^ and cancer^23^. In GO, proteomics approaches have been applied to compare the protein profiles of tears from GO patients with healthy controls^24,25^, GD patients free of eye disease^26^, smokers^27^ and people with dry-eye syndrome^28^. At present, there are no reports of proteomic profiling of serum or plasma in GO patients and the only investigation of circulating miRNA has focussed on non-responders to glucocorticoid therapy^29^. Hence, we aimed to apply high throughput proteomics and analysis of miRNA in serum to search for biomarkers that can be used to predict GO and plan the most appropriate treatment regimen. Together with the analysis of differential expression in patients and healthy controls, blood miRNAs and proteins data have also been used to predict which group (either GD, GO, healthy controls) the individual samples belonged to. By complementing results from differential expression and the predictive model, robust candidate biomarkers have been identified.

## Methods

### Ethics statement

All samples were collected between October 2014 and June 2016 within the framework of the EU-FP7 Indigo project (http://www.indigo-iapp.eu/): appropriate local research ethical approval and written informed consent was obtained from all participants.[Essen: Ethik-Kommission reference 14-5965-BO; Cardiff: Wales Research Ethics reference 12/WA/0285; Milan: Comitato Etico Milano Area B, approval obtained on 11/11/2014]. All subjects were treated in accordance with the tenets of the Declaration of Helsinki. The INDIGO study enrollment criteria were: (i) newly diagnosed or relapsing GD patients under anti thyroid treatment within six weeks from the onset of hyperthyroidism, (ii) GD patients at euthyroidism, (iii) newly diagnosed GD patients with overt GO.

### Experimental samples

From a larger cohort of patients and healthy controls recruited by the INDIGO project, 46 caucasian females (33 cases, 13 controls) were selected for miRNA and proteomics sequencing. Cases were further subdivided into Graves’ disease patients with (GO = 19) and without (GD = 14) orbitopathy. Patients were treatment naive or within 6 weeks of commencing antithyroid drug therapy. The clinical activity score of GO was assessed following EUGOGO guidelines^30^. Healthy controls were all free of thyroid disease, euthyroid and negative for TRAB. Cases and controls were recruited in three medical centres from different European countries: Cardiff University Hospital (United Kingdom), Essen University Hospital (Germany), and University of Milan (Italy). As far as possible, cases and controls were matched by age to limit external confounders. Table 1 summarizes the characteristics of the patients and controls used. Blood samples were collected from all subjects enrolled either in EDTA-coated test tubes for plasma or gel-filled test tubes for serum separation (BD Vacutainer), and processed by centrifugation at 1200 g for 15 min within one hour from collection, then stored at −20 °C until used. Samples from patients with GD and GO were obtained at the first hospital visit or when they relapsed: both conditions were diagnosed by the presence of suppressed blood TSH (thyroid-stimulating hormone), FT4 (free thyroxine) levels above the local reference range, TRAB positivity, and diffuse goitre (when present).

**Table 1:** Recruiting centre, disease status, age (years), smoking habit and mean serum biochemical parameters of patients and controls enrolled for this study (all females, all caucasians).

Centre	Status^a^	N	orbitopathy^b^	age	smokers^c^	TSH (mIU/L)	TRAB (IU/L)	FT4 (pmol/L)
Cardiff	control	4		53 ± 21.1	0	0.92	0.00	14.62
Cardiff	GD	5		51.6 ± 17.9	3	0.02	9.68	23.64
Cardiff	GO	6	4/2/0	61.3 ± 16.1	2	0.67	6.60	19.36
Essen	control	5		47.2 ± 11.7	1	2.00	0.30	12.70
Essen	GD	5		40.8 ± 10.9	3	0.01	12.71	33.12
Essen	GO	8	1/3/4	48.4 ± 10.6	6	0.32	11.41	17.96
Milano	control	4		50.2 ± 2.2	0	1.50	0.40	11.00
Milano	GD	4		57.5 ± 17.1	0	0.01	10.72	30.13
Milano	GO	5	2/3/0	47.2 ± 14.8	1	0.45	16.25	17.36

^a^GD: Graves’ disease; GO: Graves’ orbitopathy; ^b^Orbitopathy: N = number of mild/moderate/sight-threatening GO cases; ^c^smokers were defined as current or previous smokers.

### microRNA sequencing

miRNAs were extracted from plasma samples using NucleoSpin miRNA Plasma kit, following manufacturer’s instructions. Quantification and integrity of the nucleic acid were assessed with NanoDrop 1000 spectrophotometer (Thermo Scientific) and Agilent 2200 TapeStation system (Agilent Technologies), respectively, to check the requirements for library preparation. For each sample library, 5 μL of extracted small RNA were used in all the experimental procedures. Each library was prepared with the Truseq SmallRNA kit (Illumina Inc., USA) with minor modifications to the manufacturer’s protocol. In order to minimize primer dimers formation, small RNA was introduced into half of the TruSeq Small RNA Sample reagents followed by 15 cycles of PCR to amplify the library. 10 μL of unique indexed libraries was pooled and resolved on a Pippin Gel cassette 3% Agarose Dye free (BluePippin, Sage Science, MA, USA). DNA fragments from 140−160 bp (the length of miRNA inserts plus the 3′ and 5′ adaptors) were recovered in 40 μL of Pippin elution buffer and then purified by Qiagen MinElute PCR Purification kit (Qiagen, CA, USA). The indexed libraries were quantified with ABI9700 qPCR instrument using the KAPA Library Quantification Kit in triplicates, according to the manu-facturer’s protocol (Kapa Biosystems, Woburn, MA, USA). 10 μL of the pooled library at a final concentration of 2 nM were used for sequencing using Illumina HiSeq. 2000 with a 50 Single-Read sequencing module in a total number of 4 lanes.

### Nano LC/QTOF proteomic mass-spectrometry

Serum samples (35 μL) were processed through the ProteoExtract® Albumin/IgG removal kit (Calbiochem®) according to manufacturer instructions, before proteomic analyses. Protein content was next determined in the eluted extracts through the Bio-Rad Protein Assay kit (Bio-Rad, Hercules, CA, USA) with bovine *γ*-globulin as standard. Then, 50 μg proteins were loaded in 4−20% TGX Stain-Free TM precast mini gel (Bio-Rad) and subjected to SDS-PAGE electrophoresis (Bio-Rad MiniPROTEAN® Tetra Cell) to achieve a rough fractionation. The lanes from each sample were manually cut into four slices of the same size, and proteins were reduced with DTT, alkylated with iodoacetamide and overnight digested using trypsin (Promega, Madison, WI, USA). Tryptic peptides were analysed using nanoscale liquid chromatography coupled to a hybrid quadrupole-time-of-flight (Q-TOF) mass spectrometer, as previously reported^31^. With this aim, a nano LC Agilent 1260 Chip Cube source and an Agilent 6550 IFunnel Q-TOF mass spectrometer (Agilent Technologies, Santa Clara, CA, USA) were used. Briefly, peptides were enriched onto a C18 trapping column and backflush eluted into a 150 mm separation column (ProtID chip, Zorbax 300SB-C18, 5 μm pore size). An acetonitrile gradient (from 3 to 70% v/v in 50 min, then hold for 15 min) in 0.1% (v/v) formic acid was adopted for elution, with mobile phase flowing at 0.3 μL min^−1^. The QTOF was operated in tandem MS data-dependent acquisition and positive ionization mode; MS SCAN was done in the range from 300 to 1700 m/z, with 4 spectra *s*^−1^. A maximum of 20 precursors per cycle were selected for tandem MS fragmentation, using precursors absolute threshold of 1000 and a relative threshold of 0.001. MS/MS spectra of peptides were used for protein inference via database searching in Spectrum Mill MS Proteomics Workbench (Rev B.04; Agilent Technologies). Carbamidomethylation of cysteine was set as a fixed modification while trypsin selected as enzyme for protein digestion, accepting two missed cleavages per peptide. Inference was set with the following parameters: Scored Peak Intensity (SPI) ≥ 50%, precursor mass tolerance of ±10 ppm and product ions mass tolerance of ±20 ppm. The search was conducted against the proteome of *Homo sapiens* (UniProt, downloaded July 2016). The database was concatenated with the reverse one and 1% false discovery rate was selected for validation purposes. Finally, a label-free quantitation was carried out using summed peptide abundance.

### Bioinformatics processing and data normalization

Fastq files resulted from miRNA sequencing were first checked for sequence quality in terms of GC content, duplication level, length and Phred score. Reads were trimmed on base-call quality (Phred score >15 over a 4 bps sliding-window; this gives a probability lower than 0.03 that a base is called incorrectly; see Ewing *et al*.^32^ for details on Phred) and length >15 bps, as miRNAs usually have length in the range 20−22 bps. Adapters used for reverse transcription and amplification were also trimmed off the reads. Trimmed reads were used along with the human reference genome (assembly GRCh38) and a database of known mature miRNA and precursors to detect known and novel miRNAs in each sample. miRNA detection and quantification was carried out using the miRDeep2 algorithm^33^; both novel and known miRNAs were quantified in terms of counts per sample. All miRNA bioinformatics processing was streamlined using an informatic pipeline developed in-house at the Bioinformatics Unit of PTP Science Park (www.ptp.it).

RNA sequencing is known to be highly variable across samples, and normalization for library size (the total amount of sequences per sample) is therefore recommended before analysing count data. miRNA raw counts were converted within each sample to counts-per-million (CPM)^34^:

1 $$CP{M}_{i}=\frac{{X}_{i}}{N}\cdot {10}^{6}$$

where, for each sample, *CPM*_*i*_ and *X*_*i*_ are, respectively, the counts-per-million and raw counts for miRNA *i*, and *N* is the total number of reads sequenced in the sample. Only miRNAs with *CPM* ≥ 2 in more than two samples were retained for subsequent analyses.

Raw protein abundances from mass-spectrometry were normalized through log-transformation:

2 $$f({x}_{i})=\{\begin{array}{ll}0 & {\rm{if}}\,{x}_{i}=0\\ log({x}_{i}) & {\rm{if}}\,{x}_{i} > 0\end{array}$$

where *x*_*i*_ is the protein raw abundance, and *log*() is the Napierian logarithm^35^. Only proteins expressed (*x*_*i*_ > 0) in more than two samples were retained for subsequent analyses.

### Cluster analysis

Metric multidimensional scaling (i.e. Principal Coordinate Analysis^36^) of dissimilarity matrices based on miRNA and proteomics data was used to visualize how GD/GO patients and controls clustered in a Cartesian coordinate system. Euclidean distances between samples were estimated based on normalized and filtered: (i) miRNA counts, (ii) protein abundances, and (iii) miRNA counts and protein abundances combined together. Distances matrices were scaled and centered and their eigenvalues used to order principal coordinates by the proportion of variability they captured. The clustering obtained by the three distance matrices was compared.

### Differential expression of miRNAs and proteins

To detect miRNA differential expression and protein differential abundance in blood samples between the three groups (GD, GO, controls), two different models were fitted for miRNA and protein data separately. miRNA counts were modeled by a negative binomial (NB) distribution to account for overdispersion relative to a one-parameter Poisson distribution. The following generalized linear model with log-link function was fitted:

3 $$log({y}_{ijqk})={\mu }_{i}+statu{s}_{j}+centr{e}_{q}+{e}_{ijqk}$$

where *y*_*ijqk*_ are miRNA *i* counts for individual *k* with status *j* from centre *q*. Residuals -and observations- are negative-binomially distributed deviates as ${z}_{ik} \sim NB({\mu }_{i},{\sigma }_{i}^{2})$, with *μ*_*i*_ the average expression for miRNA *i*, and ${\sigma }_{i}^{2}$ its variance, given by *μ*_*i*_(1 + *ϕ*_*i*_*μ*_*i*_), where *ϕ*_*i*_ is the miRNA-specific dispersion parameter (when *ϕ* = 0, the NB distribution reduces to Poisson). Protein relative abundances were analysed through a multinomial logarithmic regression model, where the probability of individual samples to belong to any of the three classes (GD, GO, control) was modeled as:

4 $$P(Y=k|X)=\frac{{e}^{{\eta }_{k}}}{{\sum }_{l\mathrm{=1}}^{K}{e}^{{\eta }_{l}}}$$

where *P*(*Y* = *k*|*X*) is the probability of sample *Y* to belong to class *k* given its vector of features *X* (log-transformed protein abundances and centre of origin), and *η*_*k*_ is a linear predictor of the form *μ*_*k*_ + *β*_1*k*_*Centre* + *β*_2*k*_ *protein*, with class-specific mean and regression coefficients for the centre and any protein *i*. A generalized linear model with log-link function was used to fit the data:

5 $$logP(Y=k|X)={\eta }_{k}-log\sum _{k^{\prime} \in K}{\eta }^{{\eta }_{k^{\prime} }}$$

where $log{\sum }_{k^{\prime} \in K}{\eta }^{{\eta }_{k^{\prime} }}$ is a normalization constant to ensure that results will form a probability distribution (i.e. sum to one). Both for Equations (3) and (5) the likelihood ratio test between the full models and reduced models without the “status” and “protein” terms, respectively, was performed to assess significance for any tested miRNA or protein. The false discovery rate (FDR^37^) was used to monitor false positives, expressing results in terms of the empirical Bayesian probability that the null hypothesis is true conditional on the observed p-value (*P*(*H*_0_ = TRUE|p-value)).

The power of differential expression analysis depends on the sample size, the depth of sequencing and the magnitude of the differential expression between groups. Following Hart *et al*.^38^, the sample size (*n*) necessary to achieve a pre-specified statistical power of analysis can be calculated from the equation below:

6 $$n=2\cdot {({z}_{1-\frac{\alpha }{2}}+{z}_{\beta })}^{2}\frac{(\frac{1}{\mu }+{\sigma }^{2})}{(lo{g}_{e}{{\rm{\Delta }}}^{2})}$$

where ${z}_{1-\frac{\alpha }{2}}$ and *z*_*β*_ are the Gaussian deviates corresponding to the desired false positive (*α*) and false negative (*β*) rates; *μ* is the average coverage (depth of sequencing); *σ* is the coefficient of variation of the number of reads across groups; Δ is the (expected) differential expression (i.e. fold-change); and *log*_*e*_ is the Napierian logarithm. In the case of miRNAs, it is difficult to speak strictly of “coverage”; however, sequencing protocols for small RNA suggest the target of 1–2 million usable miRNA reads per sample, which can be achieved by sequencing 10 million reads per sample on average. This is what was done in the present study, giving a coverage of approximately 10. The estimated coefficient of variation of sequences across samples was 0.23 for miRNA and 0.35 for proteins. For differentially expressed genes with a fold-change of 2, Equation 6 would give a sample size between 4 and 8 individuals per group for a false positive rate (*α*) of 0.05 and a power (*β*) in the range 0.7−0.9.

### ‘omics predictive models

microRNA counts and protein relative abundances were also used for the classification problem of predicting whether individuals, based on their profiles, belonged to the GD, GO or control class. Multinomial logistic regression was used to model the posterior probabilities of the three classes *P*(*Y* = *k*|*X*), as in Equations (4) and (5). Three models were tested: (i) miRNA counts only; (ii) protein abundances only; (iii) miRNA and protein data combined. Unlike in the differential expression analysis, here miRNA and proteins were not analysed separately, one by one, rather included all together in the feature matrix *X*. The effect of centre of origin (Cardiff, Essen, Milan) was also included, in all three models. Since the number of parameters *p* (miRNA, proteins, miRNA + proteins) was always much larger than the number of individuals *n*, an *L*_1_ penalization was added to the log-likelihood function used to solve the model:

7 $$\ell ({\{{\theta }_{k}\}}_{1}^{K})=-[\frac{1}{N}\sum _{i\mathrm{=1}}^{N}(\sum _{k=1}^{K}{y}_{il}({\eta }_{k})-\,\mathrm{log}(\sum _{k\mathrm{=1}}^{K}{e}^{{\eta }_{k}}))]+\lambda [\sum _{j\mathrm{=1}}^{p}\Vert {\theta }_{j}\Vert ]$$

where *θ* is the vector of parameters (coefficients) over the *K* = 3 classes; *N* is the sample size, *y*_*il*_ = *I*(*k*_*i*_ = *l*) is an indicator variable specifying to which class the observation *y*_*i*_ belongs; *λ* is the penalization hyperparameter -to be tuned- that controls the degree of regularization. Equation 7 was solved with a partial-Newton algorithm via coordinate descent^39^.

*L*_1_-penalized Lasso (least absolute shrinkage and selection operator) models, besides allowing for solutions of $p\gg n$ problems, have the property of selecting variables (miRNA and proteins, in our case) to be included in the model, by setting some of the coefficients exactly equal to zero. On one hand, this makes matrix *X* sparse, thus speeding up computation; on the other hand, it natively returns a list of relevant predictors (variable included in the predictive model). To estimate the predictive ability of the tested Lasso-penalized multinomial logistic regression models, data were first split into a test set (1/10 of the data), where accuracy was measured, and a training set (9/10 of the data), where the model was trained, i.e. the coefficients estimated. In the training set, the *λ* Lasso-penalization coefficient was tuned through 10-fold cross-validation^40^. The final model from cross-validation was applied to the test set, and the overall and per-class error rates were obtained. The whole process was repeated 150 times, each time randomly resampling new test and training sets, in order to obtain robust estimates of the model’s predictive ability and reliability (variability of predictions).

### Functional and pathway analysis

Candidate biomarkers identified from differential expression analysis and predictive models, have been further analysed by querying biological databases for related annotated functions and pathways. For proteomics data, UniProt protein identifiers (http://www.uniprot.org/) were converted to corresponding HGNC (Human Genome Organisation Gene Nomenclature Committee, https://www.genenames.org/) gene symbols. Candidate novel miRNA sequences were aligned against the reference human genome build 38 (GRCh38) and against the corresponding transcriptome (GRCh38, cDNA) using the Basic Local Alignment Search Tool (BLAST^41^). From annotated alignments, HGNC gene symbols were obtained. Both lists of gene symbols were then used to search for related functions and metabolic pathways. Based on genes annotated to metabolic pathways in the KEGG database (http://www.kegg.jp), an enrichment analysis for the identified candidate biomarkers was conducted, to detect pathways significantly associated with the corresponding genes (protein-coding genes and miRNA targets). This was determined based on a hypergeometric distribution, on the underlying assumption of no association (random distribution of genes and pathways):

8 $$p=1-\sum _{i=0}^{k-1}\frac{(\frac{M}{i})(\frac{N-M}{n-i})}{(\frac{N}{n})}$$

where *p* is the p-value for the association between any specific pathway and the candidate biomarkers gene-set; *N* and *M* are the total number of annotated genes, and total number of genes annotated to any specific pathway, respectively, in the KEGG database; *n* is the size of the candidate-biomarkers list of genes; *k* is the number of candidate-biomarkers genes annotated to any specific pathway. The summation refers to the probability of randomly observing up to *k*−1 genes annotated to any specific pathway (the hypergeometric cumulative distribution function), whose complement to one gives the probability of not observing, by chance alone, *k* genes associated to that pathway (the p-value).

### Software

Quality check of FastQ files from miRNA sequencing was performed using the FastQC software^42^; to trim miRNA reads the programme Trimmomatic^43^ was used, while the Perl programme suit miRDeep2, developed at the Max Delbrück Center for Molecular Medicine (https://www.mdc-berlin.de/8551903/en/), was used to align and count known and novel miRNAs. The R packages edgeR^44^ and qvalue^45^ were used for differential expression analysis and the estimation of FDR; the R package ggplot2^46^ was used for all figures. BLAST 2.2.26 for Linux was used for sequence alignments to find miRNA targets. The R environment for statistical programming and Python were used for data processing and manipulation and for accessory data analysis.

### Data availability

The datasets generated by the current study are available from the corresponding author upon request.

## Results

### Analysis of sequenced miRNA and proteins

We obtained 28 GB of Fastq miRNA files demonstrating overall good sequencing quality (FastQC). On average, 13069923 reads per sample were sequenced, with an average length of 23.31 bps. After trimming (with parameters: Phred >15, length >15 bps, adapters), 52.4% of the reads were retained. Querying the miRDeep2 database with trimmed reads, and alignment against the human reference genome, yielded a total of 3025 miRNAs (1881 known, 1144 novel). Filtering for *CPM* ≥ 2 in more than two samples left 1332 miRNA available for subsequent analyses (777 known, 555 novel). From proteomic mass-spectrometry, 1886 proteins were detected which reduced to 831 proteins retained for analyses after filtering for proteins expressed in >2 samples.

### Cluster analysis

**Figure 1:**
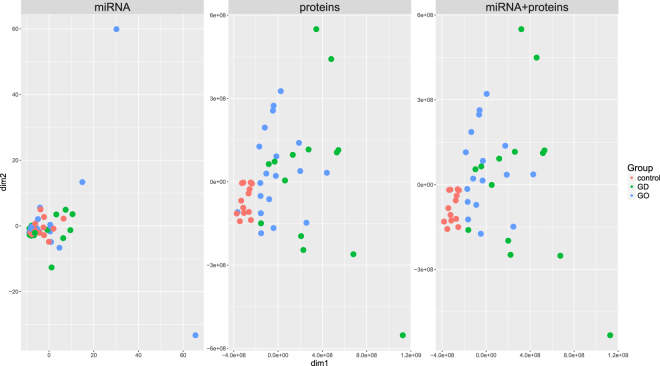
Multidimensional scaling (MDS) plots of distance matrices based on miRNA counts (left), proteins abundances (centre), miRNA and proteins together (right) from blood samples taken from Graves’ disease and orbitopathy patients and healthy controls. dim1, dim2: the first two dimensions from MDS.

Distances between samples (GD, GO and controls) were estimated from miRNA counts and protein relative abundances. Three Euclidean distance matrices were compared: based on miRNA, proteins and miRNA + proteins combined. Clearly separate clusters were not obtained from miRNA counts alone, while protein abundances and, especially, proteins and miRNA data combined did form separate clusters (77.3% of total variation accounted for by the first two dimensions from multidimensional scaling). In particular, controls were clearly distinguished from Graves’ disease cases, while a less clear boundary between GD and GO patients was obtained (Fig. 1).

### Differential expression analysis

We demonstrated 27 miRNAs (12 known, 15 novel) and 178 proteins with differential expression (FDR < 0.05) between GD, GO and control groups, as reported in Supplementary Table 1. Highest fold changes were observed between GD and controls (average 1.69), followed by GO vs controls (average 1.36), with least differential expression between GD and GO patients (average 1.01). The top known and novel miRNAs were hsa-mir-497, hsa-mir-320b-1 and hsa-mir-320b-2, up-regulated in GD, Novel:19_15038 and Novel:hsa-miR-27a-3p up-regulated in GO, and Novel:hsa-miR-22-3p which was down-regulated in GD and GO. Three of the four most differentially expressed proteins were up-regulated in GD (haptoglobin-related protein, haptoglobin–zonulin–and zinc-alpha-2-glycoprotein), except the coiled-coil domain-containing protein 25 which was down-regulated in GD and GO. Immunoglobulins (n = 19), kinases (n = 12) and complement-related proteins (n = 12) had the highest frequency among the 155 proteins with FDR < 0.05.

### Classification accuracy

**Figure 2:**
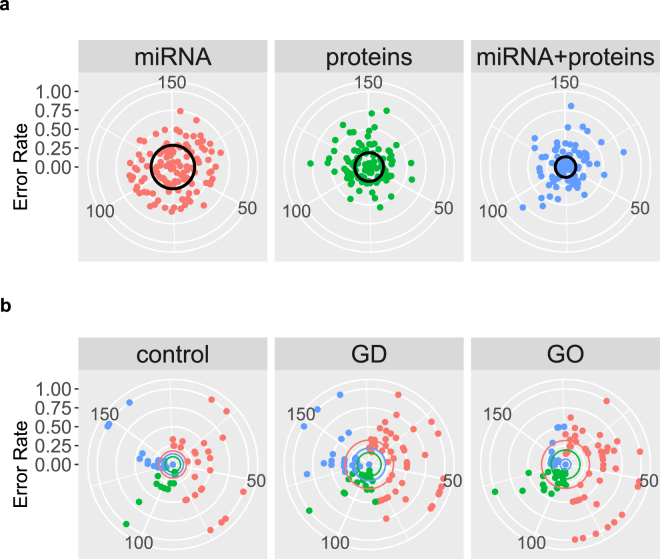
Error rates (1-accuracy) from ‘omics predictive models. (**a**) Total error rate with the three models (miRNA, proteins, miRNA + proteins); (**b**) Per-class (controls, GD, GO) error rates with the three models (different sections of the target). Black (**a**) and coloured (**b**) circles indicate average error rates, per model (above) and per class (below). Colours of the average error rate in the below graphs represent the models (miRNA, proteins and miRNA + proteins).

Three ‘omics lasso-penalized multinomial logistic regression models were compared for the classification of individual samples into the three classes (GD, GO, controls): (i) a model based only on blood miRNA counts; (ii) a model based only on blood protein relative abundances; (iii) a model based on combined blood miRNA and proteins data. The accuracy of classification is reported in Table 2, overall and per class. The error rate (1-accuracy) of the three models, either overall or per-class, is shown in Fig. 2: the closer to the centre the points lie, the better the predictive ability of the model. The spread around the centre of the target indicates the variability of predictions over the 150 replicates of each model: the less variable, the more reliable. In general, all models behaved well in terms of predictive ability, with overall average accuracy in the range 0.712−0.863. However, the average classification accuracy clearly increased when miRNA and proteins were used together in the model, and the standard deviation decreased (more reliable predictions). To ensure that overall accuracy was not driven by one of the three classes, the within-class accuracy was also evaluated. In general classification, models predicted equally well samples belonging to all classes. Predictions based on miRNA alone were substantially more variable (standard deviation 0.303, 0.337 and 0.341 in controls, GD and GO, respectively). Models based on proteins and, especially, the proteins and miRNA combined, produced higher and more stable prediction accuracy in all classes. In particular, the miRNA + proteins model had an average error rate of 7.2% in GO patients.

**Table 2:** Classification accuracy (total and per class) from Lasso-penalized logistic regression models based on miRNAs and proteins separately, and on miRNAs and proteins combined.

label	model	accuracy	std
all	miRNA	0.71	0.21
all	proteins	0.81	0.19
all	miRNA + proteins	0.86	0.18
control	miRNA	0.82	0.30
control	proteins	0.90	0.21
control	miRNA + proteins	0.86	0.27
GD	miRNA	0.68	0.34
GD	proteins	0.84	0.21
GD	miRNA + proteins	0.78	0.30
GO	miRNA	0.68	0.34
GO	proteins	0.81	0.22
GO	miRNA + proteins	0.93	0.14

The standard deviation of classification accuracy (over the 150 repetitions of each predictive model) is also reported.

Lasso models natively operate variable selection: this means that in each of the 150 replicates of the three ‘omics models, a subset of miRNA, proteins or a mix of miRNA and proteins was selected for classification. This way, the proportion of times each predictor (miRNA or protein) was selected over the 150 replicates could be calculated. Supplementary Table 2 reports all predictors used by the three models, along with their frequency. In total, 127 miRNAs, 87 proteins, and 90 between miRNA (45) and proteins (45) were used by the miRNA, proteins and miRNA + proteins models. Frequencies > 90% were obtained for three miRNAS (Novel:19_15038 (0.98), Novel:hsa-miR-6748-3p (0.98), hsa-mir-887 (0.97)) and two proteins (haptoglobin (zonulin): 0.99) and alpha-2-macroglobulin (0.99)).

### Biomarker discovery

Results from differential expression and classification models were combined together to detect robust potential blood biomarkers for GD and GO. Five sets of candidate biomarkers were considered: results from differential expression analysis of miRNA and proteins with FDR lower than 5% (mirDE and protDE), 20 top-ranked predictors -in terms of frequency of inclusion- each for the miRNA, proteins and miRNA + proteins classification models (mirPRD, protPRD, mir + protPRD). The intersections of these sets were combined in a single list of potential biomarkers as from the set expression below:

9 $$({\rm{mirDE}}\cap {\rm{mirPRD}})\cup ({\rm{protDE}}\cap {\rm{protPRD}})\cup ({\rm{mir}}+{\rm{protPRD}}\cap ({\rm{mirDE}}\cup {\rm{protDE}}))$$

**Figure 3:**
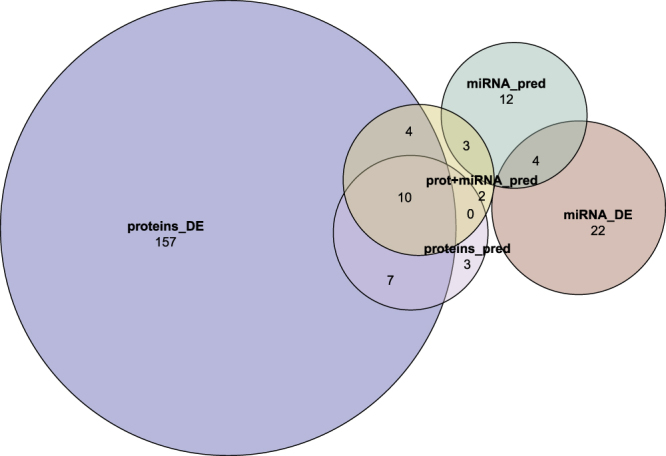
Euler Diagram of candidate biomarkers (miRNAs and proteins) detected with different approaches. mirDE, protDE: differential expression analysis for miRNAs and proteins. mirPRD, protPRD, mir + protPRD: predictive models based on miRNAs, proteins and miRNAs plus proteins (plus covariates). The intersections (N:1 + 4 = 5; 10 + 7 = 17; 10 + 1 + 4 = 15) provided the biomarkers selected for follow-up functional analysis in this paper: the hypothesis is that such biomarkers, identified consistently across different approaches, should be the most robust results.

**Figure 4:**
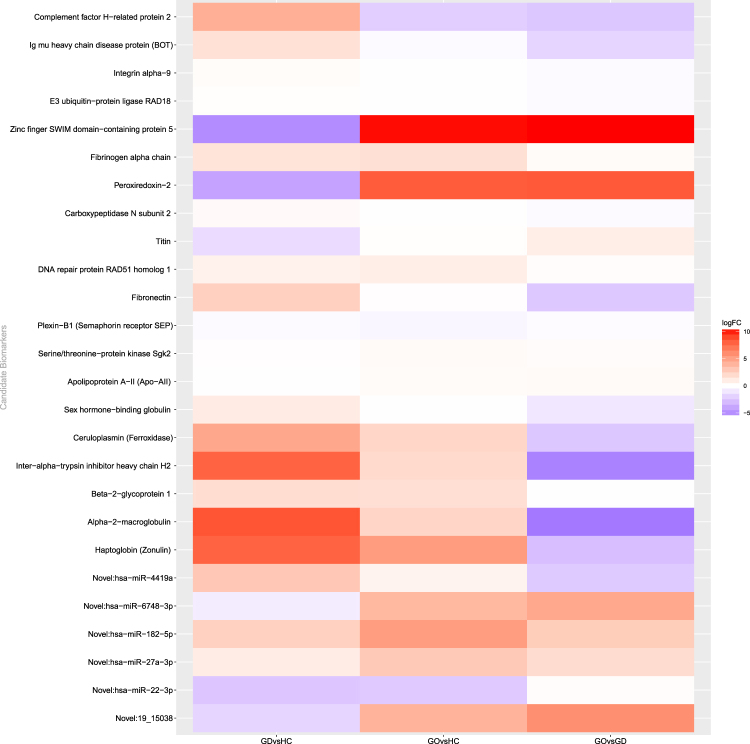
Heatmap of the *log*_2_ Fold Change (logFC) of candidate robust biomarkers listed in Table 3. Across group-comparisons: GD vs. controls (HC), GO vs. HC and GO vs. GD. logFC values are taken from differential expression (DE) analysis. Change in colour intensity refers to change in the expression of biomarkers in the three comparisons: red, positive logFC, up-regulation; blue, negative logFC, down-regulation.

The Euler diagram of the five sets of candidate biomarkers is reported in Fig. 3. Five miRNA (mirDE ∩ mirPRD), 17 proteins (protDE ∩ protPRD) and a mix of 15 miRNA and proteins (mir + protPRD ∩ (mirDE ∪ protDE)) were in common between results from differential expression and Lasso classification models. Twenty-five out of these 37 biomarkers were unique (non-duplicated), and provided the list of robust biomarkers for Graves’ disease and Graves’ orbitopathy detected in this work (Table 3). Figure 4 shows the heatmap of pairwise *log*_2_ fold-change (logFC) for the identified biomarkers listed in Table 3.

**Table 3:** Robust blood biomarkers for GD and GO identified from differential expression and classification models.

genes	qvalue	proportion	notes
Novel:19_15038	5.96e-15	0.98	novel
Novel:hsa-miR-182-5p	4.66e-04	0.59	novel
Novel:hsa-miR-22-3p	1.69e-11	0.95	novel
Novel:hsa-miR-27a-3p	9.53e-06	0.26	novel
Novel:hsa-miR-6748-3p	2.04e-03	0.98	novel
O43157	4.94e-04	0.59	Plexin-B1
P00450	3.31e-05	0.13	Ceruloplasmin
P00738	5.39e-08	0.99	Haptoglobin
P01023	6.90e-07	0.99	Alpha-2-macroglobulin
P02652	2.10e-03	0.76	Apolipoprotein A-II
P02671	1.23e-02	0.19	Fibrinogen alpha chain [Cleaved into: Fibrinopeptide A; Fibrinogen alpha chain]
P02749	2.42e-06	0.27	Beta-2-glycoprotein 1
P02751	1.02e-03	0.13	Fibronectin
P04220	1.71e-02	0.06	Ig mu heavy chain disease protein
P04278	1.41e-04	0.71	Sex hormone-binding globulin
P19823	1.24e-05	0.15	Inter-alpha-trypsin inhibitor heavy chain H2
P22792	8.11e-03	0.14	Carboxypeptidase N subunit 2
P32119	6.58e-03	0.72	Peroxiredoxin-2
P36980	3.02e-02	0.06	Complement factor H-related protein 2
Q06609	2.59e-03	0.17	DNA repair protein RAD51 homolog 1
Q13797	8.73e-03	0.07	Integrin alpha-9
Q8WZ42	6.58e-03	0.07	Titin
Q9HBY8	1.68e-04	0.87	Serine/threonine-protein kinase Sgk2
Q9NS91	3.23e-02	0.13	E3 ubiquitin-protein ligase RAD18
Q9P217	7.12e-03	0.41	Zinc finger SWIM domain-containing protein 5

These included 5 miRNA (all novel) and 20 proteins. The gene name or Uniprot identifier are reported in the first column for miRNA and proteins, respectively.

### miRNA targets

The five novel miRNAs selected as candidate biomarkers were aligned against the human genome and transcriptome to search for potential targets. miRNA molecules are known to regulate gene expression. In total, 57 target genes were detected from genome alignments, with 99.7% average sequence identity (median = 100%), and 0.29 average (median 0.16) e-value (expected number of matches to be obtained by chance alone against the human genome). From transcriptome alignments, two proteins (phosphatase and actin regulator 3; protocadherin-16) and four unannotated loci were detected, with an average identity and e-value of 100% and 0.32, respectively. Comprehensive details on miRNA targets are listed in the Supplementary Table 3.

### Metabolic pathway analysis

**Figure 5:**
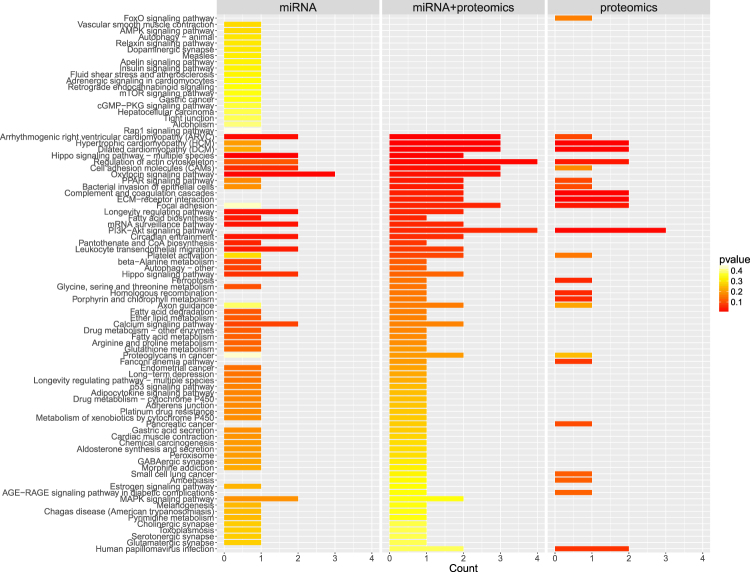
Top metabolic pathways (from KEGG) enriched in the genes associated with the candidate biomarkers for Graves’ disease and orbitopathy detected using blood proteins, blood miRNAs or both.

Pathways associated with the candidate biomarkers were identified using the KEGG (Kyoto Encyclopedia of Genes and Genomes) database of human genes and annotated metabolic pathways. They were ordered by the significance of association (Fig. 5) with the top 5 pathways for miRNA alone, protein alone and miRNA/protein combined reported in Table 4. In the combined group there were 16 pathways with p-value ≤ 0.05 (from Equation 8). These included: regulation of actin cytoskeleton, PI3K-Akt signaling pathway, Arrhythmogenic right ventricular cardiomyopathy (ARVC), Hypertrophic cardiomyopathy (HCM), Dilated cardiomyopathy (DCM), Cell adhesion molecules (CAMs), Oxytocin signaling pathway, Focal adhesion, Hippo signaling pathway, PPAR signaling pathway, Bacterial invasion of epithelial cells, Complement and coagulation cascades, ECM-receptor interaction, Longevity regulating pathway, mRNA surveillance pathway, Circadian entrainment.

**Table 4:** Top 5 pathways per analysis (miRNA, proteins, miRNA + proteins) associated with the identified biomarkers for Graves’ disease and orbitopathy (Table eftab:biomarkers).

Description	p-value	exp
Hippo signaling pathway - multiple species	0.003	miRNA
Oxytocin signaling pathway	0.008	miRNA
Arrhythmogenic right ventricular cardiomyopathy (ARVC)	0.016	miRNA
Longevity regulating pathway	0.024	miRNA
mRNA surveillance pathway	0.025	miRNA
Arrhythmogenic right ventricular cardiomyopathy (ARVC)	0.003	miRNA + proteomics
Hypertrophic cardiomyopathy (HCM)	0.005	miRNA + proteomics
Dilated cardiomyopathy (DCM)	0.006	miRNA + proteomics
Hippo signaling pathway - multiple species	0.006	miRNA + proteomics
Regulation of actin cytoskeleton	0.010	miRNA + proteomics
Complement and coagulation cascades	0.005	proteomics
ECM-receptor interaction	0.005	proteomics
Hypertrophic cardiomyopathy (HCM)	0.005	proteomics
Dilated cardiomyopathy (DCM)	0.006	proteomics
PI3K-Akt signaling pathway	0.010	proteomics

Results based on the KEGG database of human genes and metabolic pathways. Pathways are grouped per experiment (using gene symbols from proteomics, from miRNA targets, and from both proteomics and miRNA targets).

## Discussion

We present experiments aiming to define peripheral blood signatures of GD and GO based on circulating miRNAs and proteins able to discriminate between the two types of patients (GD and GO), and between affected individuals and healthy controls. The outlined approach made use of NGS ‘omics data (miRNA and proteomics), and was based on combining results from inferential and predictive statistics: differential expression analysis was conducted to understand which proteins and miRNAs are up- or down-regulated in relation with the disease; ‘omics data were used to predict to which class (GD, GO, control) individual samples belonged and, coupled with Lasso variable selection, specific proteins and miRNA most relevant for predictions could be identified. Overall, results reveal differential expression of blood miRNA and proteins between GD and GO patients and healthy controls. Biomarkers have been identified, which may be used for early diagnosis and prognosis of GD, including the likelihood of its progression to GO.

Combining results from inference and predictions, especially if leveraging data resampling techniques, is a powerful approach to detecting more robust signals of associations. In the field of genomics, combining results from GWAS and from genomic predictions has been proposed to address the issue of spurious genetic marker-phenotype associations and produce more meaningful results^47–49^ or, reverting perspective, to increase the accuracy of genomic predictions^50^. Especially if the accuracy of predictions is high, selected variables from predictive models are likely to play a role in the studied phenotype, and can effectively contribute to identifying robust biomarkers associated with the disease. When using proteins and miRNAs combined, the overall accuracy of predictions was as high as 0.86 (0.78, 0.93 and 0.86 in GD, GO and controls respectively), with little variation, which points to reliable results and to a high degree of confidence to be placed in the biomarkers detected through Lasso variable selection. Additionally, blood proteins and circulating miRNAs, with respect to GD and GO seem to be rather independent sources of information, as shown by the clear improvements obtained both in clustering and predictions when using all data together rather than proteins or miRNAs separately. However, although combining proteomics and miRNA data clearly increased the classification accuracy, the main clustering effect appeared to be played by blood proteins, with a lower role of circulating miRNA. This may be related to a larger influence of the thyroid function on the protein metabolism.

Some relevant aspects of the methodology and results are discussed below. First, the functions of the detected biomarkers are discussed and placed in the context of related literature, followed by a comparison with biomarkers for GD from previous studies. Then, the relationship between the ‘omics-based peripheral blood signature of GD and GO and the clinical features of patients is examined. Finally, the role of smoking habit as risk factor for GD is addressed.

We identified 5 miRNAs and 20 proteins combining DE and prediction models, as the most relevant biomarkers able to help distinguish either GD or GO from controls, or between GD and GO patients. These were Novel:19_15038 miRNA and Novel:hsa-miR-182-5p, both up-regulated in GO. Known miRNAs were identified by DE analysis, but not from the prediction model–although aberrant miRNA expression may go beyond being a biomarker and contribute to pathogenesis, as has been reported for cancer^23^. Amongst robustly identified proteins, Zonulin was about 8-fold and 5-fold up-regulated in GD and GO, respectively, compared to controls, and about 3-fold up-regulated in GD compared to GO. Zonulin was previously associated with impaired intestinal tight-junctions and increased gut permeability^51^, and was found to be involved in autoimmune conditions, like type 1 diabetes^52^. Could zonulin upregulation have driven our identification of the “bacterial invasion of epithelial cells” pathway? Over-expression of zonulin could impair gut permeability and lead to the presentation of environmental antigens (such as bacterial antigens) within the gut epithelial and GALT. The association between gut microbiome and thyroid autoimmunity has been recently proposed^53,54^. Celiac disease is also characterized by the presence of an impaired gut permeability; whether GD and celiac disease in this cohort of GD patients are associated is under investigation. Several fibrosis-related proteins were identified as biomarkers including Fibronectin, Plexin-B1, Fibrinogen alpha-chain, Lumican. Interestingly, alpha 2 macroglobulin and haptoglobin are known to act as markers of fibrosis in the liver, and together with ApoA-I and some key liver enzymes are being used in algorithms that stage liver fibrosis and predict its progression^55,56^. Upregulation of these protein might occur in the process leading to fibrosis of the orbital tissues in GO. Metabolic and functional pathways in which candidate biomarkers are involved, both miRNA targets and proteins, have been retrieved from biological databases. Several signaling cascades have been identified, in particular, the PI3k-Akt, mTOR, FoxO, PPAR and the adipocytokine signaling pathways^57,58^. The PI3K-Akt pathway is activated by TSH-TSHR binding and its role in promoting orbital adipogenesis in GO patients has been described^59^. The peroxisome proliferator-activated receptor (PPAR) signaling pathway was associated with thyroid-eye disease progression^60^. Several pathways associated with hypertrophic cardiomyopathy were also identified, which may be associated with the additional strain imposed on the cardio-circulatory system by hyperthyroidism.

Besides the candidate biomarkers identified by combining inferential and predictive results, additional blood proteins and miRNAs, which are known in literature to be associated with GD, were identified from either the differential expression analysis or the resampled predictive model (though not from both). Proteins identified from previous studies include *β*-2 microglobulin, proline rich protein-4, lysozyme C and zinc *α*-2 glycoprotein^26,27^. Zinc *α*-2 glycoprotein was found to be significantly differentially expressed between GD, GO and controls also in this study, with *FDR* = 5.39⋅10^−8^: in particular, it was upregulated in GD and GO patients compared to controls, less in GO than in GD patients. Baker and colleagues (2006) reported increased Zinc *α*-2 glycoprotein in the tears of GO patients and smokers compared with healthy controls and non-smokers. Another protein of interest is the lumican, a keratan sulphate proteoglycan expressed in the cornea and mesenchymal tissues throughout the body and has been reported to be associated with orbital fibrosis^61^. Higher levels of adiponectin and the sex hormone binding globulin (SHBG) in GD patients confirmed data from a previous study^62^. The hyaluronan binding protein, the retinol binding protein and the vitamin D binding protein–slightly over-expressed in GD and GO compared to controls–were, as previously reported, associated with the disease status in GD and GO. miRNA associated with Graves disease have been reported in literature^63,64^. These did not match with biomarkers identified in the present study, possibly due to the different biological tissues (PBMC -peripheral blood monocytes- vs blood) or technology (miRNA microarrays or quantitative RT-PCR).

**Figure 6:**
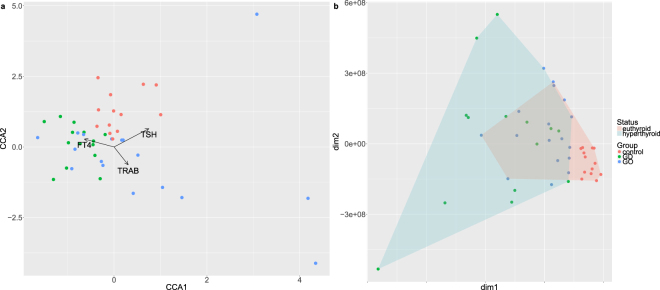
(**a**) Canonical correspondence analysis (CCA) plot of ‘omics-based Euclidean distances between samples and their quantitative clinical features (TSH, TRAB and FT4 levels). (**b**) Multidimensional scaling plot of miRNA + proteomics distances with superimposed hyperthyroidism status (euthyroid vs. hyperthyroid).

Since in most cases protein expression was higher in GD patients than GO or healthy controls, possibly reflecting their hyperthyroid status, circulating miRNAs and protein profiles were related to thyroid-receptor antibody (TRAB) titres, thyroid-stimulating hormone (TSH) and free thyroxine (FT4) concentrations, and hyperthyroid/euthyroid status using canonical correspondence analysis^65^. The hyperthyroid/euthyroid status, clinically determined and confirmed by biochemical testing, was superimposed on the distance matrix based on blood miRNA and protein counts. Results (Fig. 6) showed that GD and GO patients were hyperthyroid, and controls euthyroid; accordingly, TRAB positivity was detected in GD and GO patients. This is consistent with Graves’ disease: TRAB stimulate the thyroid to produce FT4 (and FT3) which, in turn, exert a negative feedback on TSH release from the pituitary gland. In particular, GD patients tended to be more hyperthyroid (lower TSH and higher FT4) than GO. The likely explanation is that GO patients are usually prescribed with anti-thyroid treatment promptly following the diagnosis, in order to avoid further worsening of the eye condition due to the thyroid dysfunction. It was reassuring to note that samples did not cluster entirely according to thyroid status suggesting that the differential expressions observed are also driven by immune as well as endocrine factors. This is supported by the work of Pietzner and colleagues^66^ who devised an experimental model of human hyperthyroidism and reported that several of the proteins detected in our study, e.g. lumican, fibronectin were affected by FT4, but not all e.g. zonulin.

Smoking habit is known to be related to GO^67^. Smoking was recorded from the individuals recruited for this study (Table 1): smokers were prevalently distributed in patients rather than controls (15:1). The frequency of smokers was higher in GO than in GD patients (9:6). Smoking habit appears to be quite confounded with disease status, and one may wonder whether some of the detected biomarkers are actually associated with smoking rather than the disease. Results from literature, however, show that none of the reported blood biomarkers for smoking habit^4,68,69^, nor differentially expressed lung proteins between smokers and non-smokers^70^ or genes associated with smoking from GWAS^71^, match the biomarkers for GD and GO identified in this study. This helps dispel concerns on possible confounding of detected biomarkers between Graves’ disease and smoking habit.

The study would have benefited from the inclusion of healthy controls who were smokers. Other limitations include the small number of patients and controls analyzed and the lack of a group having non-autoimmune hyperthyroidism to distinguish effects driven by immune or endocrine factors. The focus on women subjects, in addition to being relevant to the disease, also eliminated confounding effects of hormones.

To summarize, an approach combining predictive and inferential statistics was adopted for the discovery of blood biomarkers associated with Graves’ disease and orbitopathy from a miRNA and proteomics sequencing experiment. A total of 25 robust circulating biomarkers have been identified (5 novel miRNAs, 20 proteins), potentially able to discriminate between healthy controls, GD and GO patients. The biological functions of these biomarkers have also been investigated, highlighting interesting connections with fibrosis and gut permeability (and autoimmunity onset). The identified blood biomarkers may be a helpful tool for clinicians to be used for early diagnosis of Graves’ disease, and for the prognosis of the disease, including the likelihood of developing orbitopathy, and represent a step forward in the direction of technology-driven precision medicine^72^.

## Electronic supplementary material

Supplementary Table 1

Supplementary Table 2

Supplementary Table 3
