# Supplementary material for: Combining micro-RNA and protein sequencing to detect robust biomarkers for Graves’ disease and orbitopathy
Source: Sci Rep. 2018 May 30;8:8386. doi: 10.1038/s41598-018-26700-1 (PMC5976672; doi:10.1038/s41598-018-26700-1)
Supplement: Supplementary file 1 — Supplementary Table 1 [file 41598_2018_26700_MOESM1_ESM.pdf]

Combining micro-RNA and protein sequencing to detect robust biomarkers for Graves' disease and orbitopathy

Lei Zhang[1], Giulia Masetti[1,2], Giuseppe Colucci[3], Mario Salvi[3], Danila Covelli[3], Anja Eckstein[4], Ulrike Kaiser[4], Mohd Shazli Draman[1], Ilaria Müller[1], Marian Ludgate[1], Luigi Lucini[5], and Filippo Biscarini[1,6,*]

[1]Cardiff University, School of Medicine, Division of Infection & Immunity, Cardiff, UK
[2]Parco Tecnologico Padano, Bioinformatics Unit, Lodi, Italy
[3]Università degli Studi di Milano, Fondazione Ca' Granda IRCCS, Department of Clinical Sciences and Community Health, Milan, Italy
[4]University Hospital Essen/University of Duisburg-Essen, Department of Ophthalmology, Essen, Germany
[5]Università Cattolica del Sacro Cuore, Department for Sustainable food process, Piacenza, Italy
[6]CNR (National Council for Research), Institute of Biology and Biotechnology in Agriculture (IBBA), Milan, Italy

∗ Corresponding author: Filippo Biscarini; e-mail: biscarinif@cardiff.ac.uk

Supplementary Table 1: all miRNAs and proteins differentially expressed between groups (GD, GO, controls), with a FDR lower than 0.05

miRNA/protein	FDR	effectGD	effectGO	effectGO-GD	notes
Novel:19_15038	5.96E-15	-1.91891	4.09855	6.01747	novel
Novel:hsa-miR-22-3p	1.69E-11	-2.33569	-2.42860	-0.09291	novel
Novel:hsa-miR-27a-3p	9.53E-06	1.23429	2.82197	1.58769	novel
Novel:4_29251	6.96E-05	2.22772	6.52215	4.29443	novel
Novel:hsa-miR-182-5p	0.0004658244489	2.44093	4.94974	2.50881	novel
Novel:hsa-miR-1266-3p	0.002039366866	-0.00955	5.00728	5.01683	novel
Novel:hsa-miR-6748-3p	0.002039366866	-0.78436	3.46821	4.25257	novel
Novel:hsa-miR-8069	0.004767668328	-1.59079	-3.20334	-1.61255	novel
hsa-mir-320b-1	0.004804027068	2.12717	0.57032	-1.55685	known
hsa-mir-320b-2	0.004804027068	2.12757	0.56416	-1.56341	known
hsa-mir-497	0.004804027068	2.07890	0.60114	-1.47776	known
Novel:hsa-miR-4254	0.006300627671	-0.34197	2.68587	3.02784	novel
hsa-mir-10b	0.007305516756	1.58121	0.71836	-0.86285	known
Novel:7_36289	0.009429648204	3.40934	5.97773	2.56839	novel
Novel:1_16536	0.01062553255	3.22688	4.11340	0.88651	novel
Novel:hsa-miR-612	0.01090460985	6.04191	6.31219	0.27028	novel
Novel:12_6007	0.01135407236	1.26256	3.42938	2.16683	novel
hsa-mir-371a	0.01268945886	2.49467	-2.72120	-5.21587	known
Novel:hsa-miR-4731-3p	0.01268945886	-1.51550	4.00616	5.52166	novel
Novel:hsa-miR-4512	0.01679747337	1.81916	-3.36318	-5.18234	novel
hsa-mir-99a	0.01720857092	1.68092	0.55376	-1.12716	known
hsa-mir-10a	0.02650867387	1.05359	0.35305	-0.70054	known
hsa-mir-215	0.02676867914	1.53149	0.47683	-1.05466	known
hsa-mir-141	0.03112278451	1.29181	-0.37030	-1.66211	known
hsa-mir-320c-2	0.03112278451	1.86545	0.47029	-1.39516	known
hsa-mir-6131	0.03112278451	0.70625	2.76186	2.05561	known
hsa-mir-885	0.03112278451	2.27139	0.76589	-1.50550	known
P00738	5.39E-08	7.95987	5.42330	-2.62912	Haptoglobin (Zonulin)

P00739	3.54E-08	10.92844	9.32233	-1.58581	Haptoglobin-related protein
P01042	1.92E-07	5.62222	3.16028	-2.51861	Kininogen-1
P25311	5.39E-08	7.91900	5.09006	-3.04732	Zinc-alpha-2-glycoprotein
Q86WR0	5.39E-08	-0.95848	-1.16237	-0.20390	Coiled-coil domain-containing protein 25
P01023	6.90E-07	7.92032	2.29573	-4.48805	Alpha-2-macroglobulin
P04217	8.75E-07	7.69947	4.74673	-2.94985	Alpha-1B-glycoprotein
P02765	1.17E-06	5.39081	5.33376	0.00725	Alpha-2-HS-glycoprotein
P02749	2.42E-06	1.51192	1.43469	-0.07724	Beta-2-glycoprotein 1
P01009	5.48E-06	5.52784	3.56333	-1.94948	Alpha-1-antitrypsin
P02787	7.21E-06	5.55676	4.07970	-1.53104	Serotransferrin
P51884	7.49E-06	3.08760	0.41205	-2.67663	Lumican
P19823	1.24E-05	8.55083	2.04021	-6.51466	Inter-alpha-trypsin inhibitor heavy chain H2
P20742	1.33E-05	5.56701	0.81383	-4.88302	Pregnancy zone protein
P02750	1.67E-05	3.02183	0.77024	-2.24619	Leucine-rich alpha-2-glycoprotein
P10909	2.87E-05	3.10848	3.44971	0.31143	Clusterin
P02763	2.99E-05	4.08113	2.63443	-1.45391	Alpha-1-acid glycoprotein 1
P00450	3.31E-05	4.94617	2.27390	-2.64532	Ceruloplasmin
O14791	4.65E-05	1.49336	-0.07913	-1.57248	Apolipoprotein L1
P09871	6.25E-05	0.45948	0.21426	-0.24522	Complement C1s subcomponent
P00734	6.25E-05	2.14623	1.70808	-0.44073	Prothrombin
P08603	9.67E-05	1.45567	0.77205	-0.68346	Complement factor H
P02760	0.0001251682656	3.14513	2.39082	-0.76412	Protein AMBP [Cleaved into: Alpha-1-microglobulin
P04278	0.000140706413	1.07677	0.06579	-1.01102	Sex hormone-binding globulin
P00736	0.0001652695088	0.37559	0.28637	-0.08922	Complement C1r subcomponent
Q9HBY8	0.0001684858194	0.06681	0.27893	0.21211	Serine/threonine-protein kinase Sgk2
P02790	0.0003374161589	5.80446	4.74530	-1.02595	Hemopexin
O43157	0.0004940957433	-0.19885	-0.38301	-0.18417	Plexin-B1
Q5T9C9	0.000503296474	0.14377	-1.30278	-1.38847	Phosphatidylinositol 4-phosphate 5-kinase-like protein 1
P04003	0.0006160972717	2.25317	1.62160	-0.63009	C4b-binding protein alpha chain
P13671	0.001018199028	0.18831	0.34743	0.15912	Complement component C6
P02751	0.001018199028	2.45655	0.16369	-2.29023	Fibronectin
P01024	0.00115070228	4.42892	2.84497	-1.70997	Complement C3
P43652	0.001187924289	3.39795	2.22384	-1.17378	Afamin
P01008	0.001354475167	2.08482	1.18422	-0.90239	Antithrombin-III
P05155	0.001455476315	2.15160	2.04284	-0.10887	Plasma protease C1 inhibitor
Q99456	0.001593739762	-1.16131	-1.13299	0.02832	Keratin, type I cytoskeletal 12
P01594	0.001810293569	1.68935	1.62166	-0.06768	Immunoglobulin kappa variable 1-33
P01599	0.001810293569	1.68935	1.62166	-0.06768	Immunoglobulin kappa variable 1-17
P01607	0.001810293569	1.68935	1.62166	-0.06768	NA
P01608	0.001810293569	1.68935	1.62166	-0.06768	NA
P01609	0.001810293569	1.68935	1.62166	-0.06768	NA
P27169	0.00199510973	2.62783	0.09957	-2.53463	Serum paraoxonase/arylesterase 1
P02774	0.00199510973	2.54730	2.09247	-0.43930	Vitamin D-binding protein
P01593	0.002049161179	0.32875	0.30240	-0.02635	Immunoglobulin kappa variable 1D-33
P02652	0.002100417169	-0.02579	0.18518	0.21097	Apolipoprotein A-II
P19652	0.002516502857	1.95942	0.89613	-1.06891	Alpha-1-acid glycoprotein 2
Q06609	0.002586050437	0.77937	0.92439	0.14499	DNA repair protein RAD51 homolog 1
P02746	0.003388013593	0.28088	0.27812	-0.00275	Complement C1q subcomponent subunit B

Q6IEU7	0.003429990173	-0.11084	-0.26459	-0.15375	Olfactory receptor 5M10
Q8NGP8	0.003429990173	-0.11084	-0.26459	-0.15375	Olfactory receptor 5M1
P99999	0.003443263357	0.21902	0.17938	-0.03964	Cytochrome c
P19827	0.00351257706	3.01165	0.74931	-2.26192	Inter-alpha-trypsin inhibitor heavy chain H1
P07225	0.004988392448	1.70088	0.08425	-1.61667	Vitamin K-dependent protein S
P02753	0.005591894859	0.16328	0.26346	0.10018	Retinol-binding protein 4
P01596	0.005655754968	1.03990	0.77202	-0.26787	NA
Q8WZ42	0.006580379509	-0.87621	0.09082	0.96132	Titin
P32119	0.006580379509	-0.82588	1.65186	1.65452	Peroxiredoxin-2
P04275	0.006877297245	1.75781	1.50353	-0.25428	von Willebrand factor
Q06033	0.007035702143	0.07904	0.46521	0.38615	Inter-alpha-trypsin inhibitor heavy chain H3
O00268	0.00704805046	2.09672	-0.21494	-2.31188	Transcription initiation factor TFIID subunit 4
Q9P217	0.007117157963	-1.02508	2.05017	2.05244	Zinc finger SWIM domain-containing protein 5
P22792	0.008105488621	0.24242	0.04437	-0.19805	Carboxypeptidase N subunit 2
Q13797	0.008733974205	0.19062	-0.04889	-0.23951	Integrin alpha-9
P02656	0.008733974205	-0.09657	0.16987	0.26644	Apolipoprotein C-III
O75636	0.008733974205	1.06112	0.99322	-0.06792	Ficolin-3
P01598	0.009549723073	0.24675	-0.04164	-0.28839	NA
Q9Y3D8	0.009549723073	0.19730	0.29032	0.09302	Adenylate kinase isoenzyme 6
Q8NF91	0.009565840715	0.06894	0.18951	0.12056	Nesprin-1
Q13790	0.01002737676	0.28878	0.22577	-0.06302	Apolipoprotein F
P22352	0.01192269867	1.52278	1.73382	0.21108	Glutathione peroxidase 3
Q3KQV9	0.01192269867	1.34526	1.18455	-0.16072	UDP-N-acetylhexosamine pyrophosphorylase-like protein 1
P01861	0.01192269867	1.66812	1.31659	-0.35298	Immunoglobulin heavy constant gamma 4
P02671	0.01226118939	1.44998	1.65044	0.20113	Fibrinogen alpha chain [Cleaved into: Fibrinopeptide A; Fibrinogen alpha chain]
P19012	0.01257667383	-0.62588	-0.58301	0.04286	Keratin, type I cytoskeletal 15
P08727	0.01281132547	-0.62697	-0.58412	0.04285	Keratin, type I cytoskeletal 19
P01825	0.01281132547	0.93320	0.79695	-0.13634	Immunoglobulin heavy variable 4-59
P06331	0.01281132547	0.93320	0.79695	-0.13634	Immunoglobulin heavy variable 4-34
P13521	0.01281132547	0.27977	0.03223	-0.24754	Secretogranin-2
A8MT79	0.01366745387	1.69867	0.06585	-1.63278	NA
P02748	0.01442719805	1.60534	0.82668	-0.78225	Complement component C9 [Cleaved into: Complement component C9a; Complement component C9b]
P08185	0.01482521137	0.95155	-0.29641	-1.24630	Corticosteroid-binding globulin
O75161	0.01558255916	0.98919	0.80477	-0.18441	Nephrocystin-4
P42680	0.01580247319	0.77071	0.80668	0.03601	Tyrosine-protein kinase Tec
P02533	0.01619541997	-0.53933	-0.49480	0.04453	Keratin, type I cytoskeletal 14
P07996	0.01619541997	0.96747	0.95822	-0.00925	Thrombospondin-1
O75165	0.01619541997	1.20153	1.30881	0.10728	DnaJ homolog subfamily C member 13
P01600	0.01619541997	0.27075	0.20132	-0.06943	NA
P01610	0.01619541997	0.27075	0.20132	-0.06943	NA
P80362	0.01619541997	0.27075	0.20132	-0.06943	NA
P04220	0.01711051961	1.64074	-0.21041	-1.85260	Ig mu heavy chain disease protein
P08779	0.01731435447	-0.51343	-0.46783	0.04559	Keratin, type I cytoskeletal 16
Q04695	0.01755043306	-0.50967	-0.46523	0.04443	Keratin, type I cytoskeletal 17
P01717	0.01816404578	0.25392	0.09705	-0.15687	Immunoglobulin lambda variable 3-25

O43866	0.01854091753	0.21247	0.09053	-0.12194	CD5 antigen-like
Q14520	0.02139993884	1.69025	1.52587	-0.16438	Hyaluronan-binding protein 2
Q99457	0.0220232395	-1.00463	-0.01671	1.57000	Nucleosome assembly protein 1-like 3
P01764	0.02238522429	1.49207	1.21467	-0.27740	Immunoglobulin heavy variable 3-23
Q96CB9	0.02276772263	0.48684	-0.07301	-0.55987	5-methylcytosine rRNA methyltransferase NSUN4
P02768	0.02361233425	2.33983	0.49414	-1.88323	Serum albumin
Q96J92	0.0243542356	0.84277	0.71387	-0.12903	Serine/threonine-protein kinase WNK4
Q9H4A3	0.0243542356	0.84277	0.71387	-0.12903	Serine/threonine-protein kinase WNK1
Q9Y3S1	0.0243542356	0.84277	0.71387	-0.12903	Serine/threonine-protein kinase WNK2
Q14185	0.02457274163	-0.97755	-0.21654	0.75552	Dedicator of cytokinesis protein 1
Q9NNW5	0.02500118968	0.06832	-1.23479	-1.16050	WD repeat-containing protein 6
P0CF97	0.02535344393	0.77824	0.67926	-0.09899	Protein FAM200B
O75592	0.02544740195	-2.80280	-2.80280	-2.57444	E3 ubiquitin-protein ligase MYCBP2
Q96I59	0.02544740195	0.93777	0.90043	-0.03730	Probable asparagine–tRNA ligase, mitochondrial
P0CG04	0.02544740195	1.73465	1.55260	-0.18481	Immunoglobulin lambda constant 1
Q3B8N5	0.02544740195	-0.15416	-1.01703	-1.29771	Prospero homeobox protein 2
Q13094	0.02544740195	-2.95657	-2.95657	-2.75952	Lymphocyte cytosolic protein 2
P31260	0.02544740195	4.69171	-2.34585	-2.70000	Homeobox protein Hox-A10
Q9UKY1	0.02544740195	0.16224	-1.34495	-1.02400	Zinc fingers and homeoboxes protein 1
Q15848	0.02544740195	0.23724	0.22285	-0.01440	Adiponectin
Q92922	0.02544740195	4.62476	-2.31238	-2.68059	SWI/SNF complex subunit SMARCC1
Q96L34	0.02549663195	1.66192	1.51643	-0.14546	MAP/microtubule affinity-regulating kinase 4
P02766	0.02549663195	1.12763	1.51205	0.38463	Transthyretin
Q2M385	0.02549663195	5.16525	-2.58262	-1.04160	Macrophage-expressed gene 1 protein
B9A064	0.02624109305	1.70374	1.53116	-0.17602	Immunoglobulin lambda-like polypeptide 5
P01871	0.02631348349	0.88478	-0.92863	-1.80467	Immunoglobulin heavy constant mu
P01859	0.02716889482	1.33927	0.96727	-0.37316	Immunoglobulin heavy constant gamma 2
Q9BYV7	0.02716889482	0.70382	0.79109	0.08720	Beta,beta-carotene 9',10'-oxygenase
Q13772	0.02908632733	1.07483	0.91426	-0.16057	Nuclear receptor coactivator 4
P31749	0.02908632733	0.13690	0.17832	0.04142	RAC-alpha serine/threonine-protein kinase
P31751	0.02908632733	0.13690	0.17832	0.04142	RAC-beta serine/threonine-protein kinase
Q9Y243	0.02908632733	0.13690	0.17832	0.04142	RAC-gamma serine/threonine-protein kinase
A0M8Q6	0.02908632733	1.35469	1.70166	0.31508	Immunoglobulin lambda constant 7
Q02985	0.02908632733	0.20416	0.09863	-0.10553	Complement factor H-related protein 3
P01763	0.02908632733	-1.00742	-0.14803	1.30318	Immunoglobulin heavy variable 3-48
P01767	0.02908632733	-1.00742	-0.14803	1.30318	Immunoglobulin heavy variable 3-53

Q99550	0.02908632733	0.66856	0.75667	0.08812	M-phase phosphoprotein 9
P01860	0.02908632733	0.93930	1.06188	0.12672	Immunoglobulin heavy constant gamma 3
P03952	0.02919692198	0.18178	0.16759	-0.01419	Plasma kallikrein
Q6PKC3	0.03018625363	-0.61109	1.22258	1.22486	Thioredoxin domain-containing protein 11
P20851	0.03018625363	4.50296	-2.25148	-2.61419	C4b-binding protein beta chain
Q01955	0.03018625363	5.17219	-2.58609	-2.90158	Collagen alpha-3
Q14344	0.03018625363	5.25195	-2.62598	-2.93663	Guanine nucleotide-binding protein subunit alpha-13
P36980	0.03018625363	4.45632	-2.22816	-1.99559	Complement factor H-related protein 2
Q9UNN5	0.03018625363	5.34211	-2.67105	-1.37073	FAS-associated factor 1
Q99759	0.03018625363	5.44060	-2.72030	-1.41842	Mitogen-activated protein kinase kinase kinase 3
Q15323	0.03127045913	-0.13853	-0.20826	-0.06972	Keratin, type I cuticular Ha1
Q9NS91	0.03230311725	0.10159	-0.07326	-0.17485	E3 ubiquitin-protein ligase RAD18
Q92831	0.03295382685	-0.02889	-0.22361	-0.19471	Histone acetyltransferase KAT2B
Q8NAT1	0.03613975971	0.83081	0.89239	0.06153	Protein O-linked-mannose beta-1,4-N-acetylglucosaminyltransferase 2
P80108	0.03621391299	0.19887	0.23872	0.03986	Phosphatidylinositol-glycan-specific phospholipase D
P02647	0.03668079664	1.36754	1.35021	-0.01386	Apolipoprotein A-I
P01613	0.03831981614	0.25356	0.17155	-0.08201	NA
Q96QU1	0.03863111613	0.07916	-0.11539	-0.19455	Protocadherin-15
Q8IZF6	0.03863111613	1.34025	1.32442	-0.01588	Adhesion G-protein coupled receptor G4
P0CG06	0.03919872711	1.45208	1.56040	0.11219	NA
P01781	0.03919872711	0.98731	0.81577	-0.17154	NA
P0CG05	0.03919872711	1.44900	1.55747	0.11224	NA
P0CF74	0.04200143942	1.39680	1.58419	0.18725	Immunoglobulin lambda constant 6
Q9NQW8	0.04228107109	1.06855	0.90006	-0.16849	Cyclic nucleotide-gated cation channel beta-3
Q92764	0.04228107109	-0.11842	-0.24360	-0.12518	Keratin, type I cuticular Ha5
Q9Y2I1	0.04228107109	1.21608	1.35576	0.13970	Nischarin
P35858	0.04446224973	1.06291	0.91917	-0.14383	Insulin-like growth factor-binding protein complex acid labile subunit
P20930	0.04749758477	-0.04545	-0.77688	-1.21732	Filaggrin
P0C0L5	0.04749758477	1.63817	1.06846	-0.56669	Complement C4-B
Q16629	0.04896426683	-1.17629	-0.05372	0.65301	Serine/arginine-rich splicing factor 7
Q6UY18	0.04896426683	0.74932	0.68165	-0.06768	Leucine-rich repeat and immunoglobulin-like domain-containing nogo receptor-interacting protein 4
P0C0L4	0.04896426683	1.62460	1.03066	-0.59096	Complement C4-A
P04180	0.04896426683	0.20130	0.00606	-0.19524	Phosphatidylcholine-sterol acyltransferase
Q9Y4F4	0.04896426683	0.10231	-0.00565	-0.10796	TOG array regulator of axonemal microtubules protein 1
P01714	0.04896426683	0.20476	0.17944	-0.02532	Immunoglobulin lambda variable 3-19
P02743	0.04896426683	0.64559	1.06697	0.42153	Serum amyloid P-component
O43790	0.04896426683	-1.38997	-1.31076	-0.04576	Keratin, type II cuticular Hb6
P78385	0.04896426683	-1.38997	-1.31076	-0.04576	Keratin, type II cuticular Hb3

P78386	0.04896426683	-1.38997	-1.31076	-0.04576	Keratin, type II cuticular Hb5
Q14533	0.04896426683	-1.38997	-1.31076	-0.04576	Keratin, type II cuticular Hb1
O60706	0.04896426683	-0.86231	-0.86552	-0.04355	ATP-binding cassette sub-family C member 9
H7BZ55	0.04934574214	-0.53357	1.06784	1.07014	Putative ciliary rootlet coiled-coil protein 2
P06681	0.04934574214	0.18158	0.08800	-0.09357	Complement C2
Q8TBZ0	0.04934574214	-0.04937	-1.48182	-1.44946	Coiled-coil domain-containing protein 110
Q06730	0.04934574214	-0.92115	0.06374	0.88044	Zinc finger protein 33A
Q9UGM5	0.04934574214	0.19264	0.08359	-0.10907	Fetuin-B
Q9NTG1	0.04961990572	-0.29577	1.69783	1.13796	Polycystic kidney disease and receptor for egg jelly-related protein
O43395	0.04961990572	-0.51676	1.03460	1.03666	U4/U6 small nuclear ribonucleoprotein Prp3
