# Supplementary material for: Combining micro-RNA and protein sequencing to detect robust biomarkers for Graves’ disease and orbitopathy
Source: Sci Rep. 2018 May 30;8:8386. doi: 10.1038/s41598-018-26700-1 (PMC5976672; doi:10.1038/s41598-018-26700-1)
Supplement: Supplementary file 2 — Supplementary Table 2 [file 41598_2018_26700_MOESM2_ESM.pdf]

Combining micro-RNA and protein sequencing to detect robust biomarkers for Graves' disease and orbitopathy

Lei Zhang[1], Giulia Masetti[1,2], Giuseppe Colucci[3], Mario Salvi[3], Danila Covelli[3], Anja Eckstein[4], Ulrike Kaiser[4], Mohd Shazli Draman[1], Ilaria Müller[1], Marian Ludgate[1], Luigi Lucini[5], and Filippo Biscarini[1,6,*]

[1]Cardiff University, School of Medicine, Division of Infection & Immunity, Cardiff, UK
[2]Parco Tecnologico Padano, Bioinformatics Unit, Lodi, Italy
[3]Università degli Studi di Milano, Fondazione Ca' Granda IRCCS, Department of Clinical Sciences and Community Health, Milan, Italy
[4]University Hospital Essen/University of Duisburg-Essen, Department of Ophthalmology, Essen, Germany
[5]Università Cattolica del Sacro Cuore, Department for Sustainable food process, Piacenza, Italy
[6]CNR (National Council for Research), Institute of Biology and Biotechnology in Agriculture (IBBA), Milan, Italy

∗ Corresponding author: Filippo Biscarini; e-mail: biscarinif@cardiff.ac.uk

Supplementary Table 2: all predictors selected by the Lasso-penalized predictive models

miRNA			proteins			miRNA + proteins		
predictor	N	proportion	predictor	N	proportion	predictor	N	proportion
Novel:19_15038	147	0.9800	P00738	148	0.9867	P01023	147	0.9800
Novel:hsa-miR-6748-3p	147	0.9800	P01023	148	0.9867	P00738	146	0.9733
hsa-mir-887	145	0.9667	Q9HBY8	90	0.6000	Novel:hsa-miR-22-3p	143	0.9533
hsa-mir-128-2	114	0.7600	P32119	89	0.5933	Q9HBY8	131	0.8733
Novel:hsa-miR-22-3p	97	0.6467	P04278	78	0.5200	P02652	114	0.7600
Novel:5_32360	94	0.6267	O43157	45	0.3000	P32119	108	0.7200
Novel:hsa-miR-182-5p	89	0.5933	P02671	29	0.1933	P04278	107	0.7133
hsa-mir-5588	73	0.4867	Q9NYI0	29	0.1933	O43157	89	0.5933
hsa-mir-4732	68	0.4533	Q06609	25	0.1667	Novel:hsa-miR-4419a	75	0.5000
Novel:hsa-miR-4632-5p	67	0.4467	P19823	23	0.1533	hsa-mir-887	62	0.4133
hsa-mir-152	65	0.4333	P02751	20	0.1333	Q9P217	62	0.4133
Novel:hsa-miR-647	61	0.4067	O43559	17	0.1133	P02749	41	0.2733
Novel:1_20060	50	0.3333	Q9P217	14	0.0933	Q06609	26	0.1733
Novel:hsa-miR-4419a	50	0.3333	P02652	12	0.0800	Novel:hsa-miR-4461	22	0.1467
hsa-mir-3173	44	0.2933	Q8WZ42	11	0.0733	Novel:22_22574	21	0.1400
hsa-mir-146b	43	0.2867	O14523	10	0.0667	P22792	21	0.1400
Novel:hsa-miR-27a-3p	39	0.2600	Q06730	10	0.0667	P00450	20	0.1333
hsa-mir-363	36	0.2400	P04220	9	0.0600	Q9NS91	20	0.1333
smoker	33	0.2200	P36980	9	0.0600	P02671	15	0.1000
hsa-mir-27a	32	0.2133	Q13797	9	0.0600	hsa-mir-5588	12	0.0800
Novel:16_10501	31	0.2067	Q9Y2M0	9	0.0600	Q13797	11	0.0733
hsa-mir-139	29	0.1933	P00450	8	0.0533	Novel:5_32360	10	0.0667
Novel:hsa-miR-1249-5p	29	0.1933	P22352	8	0.0533	hsa-mir-139	9	0.0600
hsa-mir-4724	28	0.1867	Q8N228	8	0.0533	P22352	8	0.0533
hsa-mir-1246	26	0.1733	Q6MZM0	7	0.0467	hsa-mir-152	7	0.0467
Novel:1_18873	24	0.1600	Q8IZJ3	7	0.0467	O75165	7	0.0467
hsa-mir-636	23	0.1533	Q9NS91	7	0.0467	P36980	7	0.0467

hsa-mir-3125	22	0.1467	Q9Y4B5	7	0.0467	hsa-mir-128-2	6	0.0400
Novel:19_15451	21	0.1400	P22792	6	0.0400	Novel:hsa-miR-500b-3p	6	0.0400
Novel:hsa-miR-9-5p	21	0.1400	P20742	5	0.0333	P09871	6	0.0400
Novel:1_19188	20	0.1333	Q01955	5	0.0333	Novel:11_2174	5	0.0333
Novel:19_15452	20	0.1333	Q502W6	5	0.0333	P02751	5	0.0333
Novel:hsa-miR-423-5p	17	0.1133	Q5VV67	5	0.0333	Q02985	5	0.0333
hsa-mir-3909	16	0.1067	Q8N7M2	5	0.0333	Q9Y4B5	5	0.0333
Novel:hsa-miR-500b-3p	14	0.0933	Q9Y2I1	5	0.0333	hsa-mir-1283-1	4	0.0267
Novel:hsa-miR-4461	13	0.0867	O14791	4	0.0267	hsa-mir-1283-2	4	0.0267
hsa-mir-378g	12	0.0800	Q96J92	4	0.0267	Novel:hsa-miR-548av-3p	4	0.0267
hsa-mir-627	12	0.0800	Q96T58	4	0.0267	P01040	4	0.0267
hsa-mir-23b	11	0.0733	Q9H4A3	4	0.0267	P04220	4	0.0267
Novel:12_5186	11	0.0733	Q9NNW5	4	0.0267	Q14520	4	0.0267
hsa-mir-3656	10	0.0667	P02814	3	0.0200	Q9Y2I1	4	0.0267
hsa-mir-548w	10	0.0667	P19827	3	0.0200	hsa-mir-153-2	3	0.0200
Novel:hsa-miR-3929	10	0.0667	Q15323	3	0.0200	hsa-mir-181b-2	3	0.0200
hsa-mir-153-2	9	0.0600	Q6Q0C0	3	0.0200	hsa-mir-3145	3	0.0200
hsa-mir-490	9	0.0600	ex_smoker	2	0.0133	hsa-mir-363	3	0.0200
hsa-mir-6499	9	0.0600	H7BZ55	2	0.0133	Novel:hsa-miR-4632-5p	3	0.0200
Novel:10_1505	9	0.0600	P01040	2	0.0133	O60706	3	0.0200
Novel:3_28477	9	0.0600	P02753	2	0.0133	Q06730	3	0.0200
Novel:11_4162	8	0.0533	P02768	2	0.0133	Q6MZM0	3	0.0200
Novel:X_43457	8	0.0533	P35858	2	0.0133	Q6PKC3	3	0.0200
hsa-mir-187	7	0.0467	Q13094	2	0.0133	hsa-mir-27a	2	0.0133
hsa-mir-221	6	0.0400	Q6PKC3	2	0.0133	Novel:1_20060	2	0.0133
Novel:18_14389	6	0.0400	Q9H1K4	2	0.0133	Novel:19_15038	2	0.0133
hsa-mir-6807	5	0.0333	Q9Y3S1	2	0.0133	Novel:2_25833	2	0.0133
Novel:20_20638	5	0.0333	A7KAX9	1	0.0067	Q15323	2	0.0133
Novel:22_22574	5	0.0333	O00165	1	0.0067	Q8N7M2	2	0.0133
Novel:hsa-miR-3690	5	0.0333	O43790	1	0.0067	hsa-mir-146b	1	0.0067
Novel:hsa-miR-6504-3p	5	0.0333	O60706	1	0.0067	hsa-mir-23b	1	0.0067
hsa-mir-1283-1	4	0.0267	O75131	1	0.0067	hsa-mir-2467	1	0.0067
hsa-mir-1283-2	4	0.0267	O75165	1	0.0067	hsa-mir-3125	1	0.0067
Novel:15_10045	4	0.0267	O95477	1	0.0067	hsa-mir-320c-2	1	0.0067
Novel:hsa-miR-8083	4	0.0267	P01042	1	0.0067	hsa-mir-3656	1	0.0067
hsa-mir-129-1	3	0.0200	P02749	1	0.0067	hsa-mir-3688-1	1	0.0067
hsa-mir-129-2	3	0.0200	P02750	1	0.0067	hsa-mir-516b-1	1	0.0067
hsa-mir-18b	3	0.0200	P04114	1	0.0067	hsa-mir-6087	1	0.0067
hsa-mir-3620	3	0.0200	P05546	1	0.0067	hsa-mir-92b	1	0.0067
hsa-mir-4755	3	0.0200	P08519	1	0.0067	Novel:12_4982	1	0.0067
hsa-mir-7975	3	0.0200	P09871	1	0.0067	Novel:16_11443	1	0.0067
Novel:12_6225	3	0.0200	P0C6C1	1	0.0067	Novel:4_28934	1	0.0067
Novel:hsa-miR-4712-3p	3	0.0200	P20851	1	0.0067	Novel:6_35067	1	0.0067
Novel:hsa-miR-5691	3	0.0200	P43652	1	0.0067	Novel:7_36432	1	0.0067
Novel:hsa-miR-612	3	0.0200	P78385	1	0.0067	Novel:7_38094	1	0.0067
hsa-mir-10a	2	0.0133	P78386	1	0.0067	Novel:hsa-miR-1299	1	0.0067
hsa-mir-1273a	2	0.0133	Q02985	1	0.0067	Novel:hsa-miR-1538	1	0.0067
hsa-mir-146a	2	0.0133	Q14533	1	0.0067	Novel:hsa-miR-3929	1	0.0067
hsa-mir-320c-2	2	0.0133	Q5T011	1	0.0067	Novel:hsa-miR-4428	1	0.0067
hsa-mir-497	2	0.0133	Q6V0I7	1	0.0067	Novel:hsa-miR-4689	1	0.0067
hsa-mir-5188	2	0.0133	Q8IZL9	1	0.0067	O43790	1	0.0067
hsa-mir-660	2	0.0133	Q8N201	1	0.0067	P02100	1	0.0067
hsa-mir-6763	2	0.0133	Q92764	1	0.0067	P19823	1	0.0067
Novel:11_2174	2	0.0133	Q92922	1	0.0067	P19827	1	0.0067
Novel:11_3221	2	0.0133	Q99759	1	0.0067	P20742	1	0.0067
Novel:11_3956	2	0.0133	Q9BSG1	1	0.0067	P35858	1	0.0067
Novel:1_16569	2	0.0133	Q9BWF2	1	0.0067	P78385	1	0.0067
Novel:1_18710	2	0.0133	Q9BWT6	1	0.0067	P78386	1	0.0067
Novel:17_12891	2	0.0133	Q9H479	1	0.0067	Q01955	1	0.0067
Novel:6_35067	2	0.0133	Q9HCI6	1	0.0067	Q14533	1	0.0067

Novel:hsa-miR-3120-5p	2	0.0133
Novel:hsa-miR-4428	2	0.0133
Novel:hsa-miR-4512	2	0.0133
Novel:hsa-miR-548a-3p	2	0.0133
Novel:hsa-miR-6732-5p	2	0.0133
Novel:hsa-miR-875-3p	2	0.0133
hsa-mir-1193	1	0.0067
hsa-mir-181b-2	1	0.0067
hsa-mir-2467	1	0.0067
hsa-mir-3122	1	0.0067
hsa-mir-3196	1	0.0067
hsa-mir-337	1	0.0067
hsa-mir-3688-1	1	0.0067
hsa-mir-4670	1	0.0067
hsa-mir-516b-1	1	0.0067
hsa-mir-548av	1	0.0067
hsa-mir-548y	1	0.0067
hsa-mir-6804	1	0.0067
hsa-mir-6865	1	0.0067
Novel:11_3715	1	0.0067
Novel:12_4613	1	0.0067
Novel:16_11142	1	0.0067
Novel:17_12406	1	0.0067
Novel:19_14927	1	0.0067
Novel:20_21294	1	0.0067
Novel:21_21587	1	0.0067
Novel:3_27858	1	0.0067
Novel:5_32796	1	0.0067
Novel:6_34961	1	0.0067
Novel:7_36479	1	0.0067
Novel:hsa-miR-1298-3p	1	0.0067
Novel:hsa-miR-1299	1	0.0067
Novel:hsa-miR-3620-5p	1	0.0067
Novel:hsa-miR-4767	1	0.0067
Novel:hsa-miR-518a-5p	1	0.0067
Novel:hsa-miR-5193	1	0.0067
Novel:hsa-miR-548av-3p	1	0.0067
Novel:hsa-miR-548u	1	0.0067
Novel:hsa-miR-6784-5p	1	0.0067
Novel:hsa-miR-6792-5p	1	0.0067

Q9NNW5	1	0.0067
Q9UBR1	1	0.0067
Q9UKY1	1	0.0067
