# Supplementary material for: Combining micro-RNA and protein sequencing to detect robust biomarkers for Graves’ disease and orbitopathy
Source: Sci Rep. 2018 May 30;8:8386. doi: 10.1038/s41598-018-26700-1 (PMC5976672; doi:10.1038/s41598-018-26700-1)
Supplement: Supplementary file 3 — Supplementary Table 3 [file 41598_2018_26700_MOESM3_ESM.pdf]

Combining micro-RNA and protein sequencing to detect robust biomarkers for Graves' disease and orbitopathy

Lei Zhang[1], Giulia Masetti[1,2], Giuseppe Colucci[3], Mario Salvi[3], Danila Covelli[3], Anja Eckstein[4], Ulrike Kaiser[4], Mohd Shazli Draman[1], Ilaria Müller[1], Marian Ludgate[1], Luigi Lucini[5], and Filippo Biscarini[1,6,*]

[1]Cardiff University, School of Medicine, Division of Infection & Immunity, Cardiff, UK
[2]Parco Tecnologico Padano, Bioinformatics Unit, Lodi, Italy
[3]Università degli Studi di Milano, Fondazione Ca' Granda IRCCS, Department of Clinical Sciences and Community Health, Milan, Italy
[4]University Hospital Essen/University of Duisburg-Essen, Department of Ophthalmology, Essen, Germany
[5]Università Cattolica del Sacro Cuore, Department for Sustainable food process, Piacenza, Italy
[6]CNR (National Council for Research), Institute of Biology and Biotechnology in Agriculture (IBBA), Milan, Italy

∗ Corresponding author: Filippo Biscarini; e-mail: biscarinif@cardiff.ac.uk

Supplementary Table 3: miRNA targets of blood miRNA identified as robust biomarkers for Graves' disease and orbitopathy

gene	gene_symbol	alignment	identity	evalue
Novel:hsa-miR-182-5p_11	RP11-712B9.2	genome	100	0.08
Novel:hsa-miR-182-5p_1	SMG5	genome	100	0.32
Novel:hsa-miR-182-5p_14	MAP4K5	genome	100	0.32
Novel:19_15038_20	AL121588.1	genome	100	0.003
Novel:19_15038_19	RFX2	genome	100	0.63
Novel:19_15038_12	RP11-541G9.1	genome	100	0.01
Novel:19_15038_12	RP11-654D12.2	genome	100	0.16
Novel:19_15038_12	KRT3	genome	100	0.63
Novel:19_15038_12	DAO	genome	100	0.63
Novel:19_15038_X	MSN	genome	100	0.04
Novel:19_15038_7	RPA3OS	genome	100	0.63
Novel:19_15038_6	PPIL6	genome	100	0.04
Novel:19_15038_6	FILIP1	genome	100	0.16
Novel:19_15038_6	RP3-399L15.3	genome	100	0.16
Novel:19_15038_6	EYS	genome	95.24	0.63
Novel:19_15038_5	CDH12	genome	100	0.04
Novel:19_15038_5	SH3TC2	genome	100	0.16
Novel:19_15038_5	RASGEF1C	genome	100	0.16
Novel:19_15038_5	CTD-2139B15.4	genome	100	0.63
Novel:19_15038_5	C5orf56	genome	100	0.63
Novel:19_15038_4	RP11-141E13.1	genome	100	0.04
Novel:19_15038_4	APBB2	genome	100	0.16
Novel:19_15038_4	MGST2	genome	100	0.16
Novel:19_15038_4	ENPP6	genome	100	0.16
Novel:19_15038_3	LMCD1-AS1	genome	100	0.04
Novel:19_15038_3	WWTR1	genome	100	0.04
Novel:19_15038_3	MYLK-AS1	genome	100	0.16
Novel:19_15038_2	AC114788.1	genome	100	0.04

Novel:19_15038_2	CTNNA2	genome	100	0.63
Novel:19_15038_2	CACNB4	genome	100	0.63
Novel:19_15038_1	AGBL4	genome	100	0.04
Novel:19_15038_1	PAPPA2	genome	100	0.04
Novel:19_15038_1	HIVEP3	genome	100	0.16
Novel:19_15038_1	AL136529.1	genome	100	0.63
Novel:19_15038_18	CD226	genome	100	0.04
Novel:19_15038_18	CELF4	genome	95.24	0.63
Novel:19_15038_17	MSI2	genome	100	0.04
Novel:19_15038_17	PITPNC1	genome	100	0.16
Novel:19_15038_17	RPTOR	genome	100	0.16
Novel:19_15038_8	RP11-127H5.1	genome	100	0.16
Novel:19_15038_8	SCARA3	genome	95.24	0.63
Novel:19_15038_8	DPYS	genome	100	0.63
Novel:19_15038_10	RP11-462L8.1	genome	100	0.16
Novel:19_15038_10	DLG5	genome	100	0.16
Novel:19_15038_10	GBF1	genome	100	0.16
Novel:19_15038_11	LRRC4C	genome	100	0.63
Novel:hsa-miR-6748-3p_16	CACNA1H	genome	100	0.08
Novel:hsa-miR-6748-3p_22	MN1	genome	100	0.32
Novel:hsa-miR-22-3p_9	TLE4	genome	100	0.2
Novel:hsa-miR-22-3p_16	GNAO1	genome	100	0.79
Novel:hsa-miR-182-5p_11	SESN3	genome	100	0.08
Novel:19_15038_19	ACSBG2	genome	100	0.63
Novel:19_15038_6	RP11-415D17.3	genome	100	0.16
Novel:19_15038_3	MYLK	genome	100	0.16
Novel:19_15038_16	C16orf45	genome	100	0.63
Novel:hsa-miR-22-3p_16	RP11-441F2.2	genome	100	0.79
Novel:19_15038_5	CTC-529P8.1	genome	100	0.16
Novel:hsa-miR-6748-3p	PHACTR3	transcriptome	100	0.33
Novel:hsa-miR-6748-3p	PHACTR3	transcriptome	100	0.33
Novel:hsa-miR-6748-3p	PHACTR3	transcriptome	100	0.33
Novel:hsa-miR-6748-3p	DCHS1	transcriptome	100	0.33
Novel:hsa-miR-6748-3p	PHACTR3	transcriptome	100	0.33
Novel:hsa-miR-6748-3p	PHACTR3	transcriptome	100	0.33
Novel:hsa-miR-6748-3p	PHACTR3	transcriptome	100	0.33
Novel:hsa-miR-6748-3p	PHACTR3	transcriptome	100	0.33
Novel:hsa-miR-6748-3p	PHACTR3	transcriptome	100	0.33
Novel:hsa-miR-6748-3p	PHACTR3	transcriptome	100	0.33
Novel:hsa-miR-6748-3p	PHACTR3	transcriptome	100	0.33
Novel:hsa-miR-6748-3p	PHACTR3	transcriptome	100	0.33
Novel:hsa-miR-6748-3p	PHACTR3	transcriptome	100	0.33
Novel:hsa-miR-6748-3p	PHACTR3	transcriptome	100	0.33
Novel:hsa-miR-6748-3p	PHACTR3	transcriptome	100	0.33
Novel:19_15038	LOC105376815	transcriptome	100	0.14
Novel:19_15038	LOC105376815	transcriptome	100	0.14
Novel:19_15038	LOC101929475	transcriptome	100	0.035
Novel:19_15038	LOC107985271	transcriptome	100	0.54
Novel:hsa-miR-6748-3p	LOC105374902	transcriptome	100	0.33
Novel:hsa-miR-6748-3p	LOC105374902	transcriptome	100	0.33
Novel:hsa-miR-6748-3p	LOC105374902	transcriptome	100	0.33
